# A Mutation in *MRH2* Kinesin Enhances the Root Hair Tip Growth Defect Caused by Constitutively Activated ROP2 Small GTPase in *Arabidopsis*

**DOI:** 10.1371/journal.pone.0001074

**Published:** 2007-10-24

**Authors:** Guohua Yang, Peng Gao, Hua Zhang, Shanjin Huang, Zhi-Liang Zheng

**Affiliations:** 1 Department of Biological Sciences, Lehman College, City University of New York, Bronx, New York, United States of America; 2 Key Laboratory of Photosynthesis and Environmental Molecular Physiology, Center for Signal Transduction and Metabolomics, Institute of Botany, Chinese Academy of Sciences, Beijing, China; 3 Plant Sciences PhD Subprogram, Graduate School and University Center, City University of New York, New York, United States of America; Umeå Plant Science Centre, Sweden

## Abstract

Root hair tip growth provides a unique model system for the study of plant cell polarity. Transgenic plants expressing constitutively active (CA) forms of ROP (Rho-of-plants) GTPases have been shown to cause the disruption of root hair polarity likely as a result of the alteration of actin filaments (AF) and microtubules (MT) organization. Towards understanding the mechanism by which ROP controls the cytoskeletal organization during root hair tip growth, we have screened for *CA-rop2* suppressors or enhancers using CA1-1, a transgenic line that expresses *CA-rop2* and shows only mild disruption of tip growth. Here, we report the characterization of a *CA-rop2* enhancer (*cae1-1* CA1-1) that exhibits bulbous root hairs. The *cae1-1* mutation on its own caused a waving and branching root hair phenotype. *CAE1* encodes the root hair growth-related, ARM domain-containing kinesin-like protein MRH2 (and thus *cae1-1* was renamed to *mrh2-3*). Cortical MT displayed fragmentation and random orientation in *mrh2* root hairs. Consistently, the MT-stabilizing drug taxol could partially rescue the wavy root hair phenotype of *mrh2-3*, and the MT-depolymerizing drug Oryzalin slightly enhanced the root hair tip growth defect in CA1-1. Interestingly, the addition of the actin-depolymerizing drug Latrunculin B further enhanced the Oryzalin effect. This indicates that the cross-talk of MT and AF organization is important for the *mrh2-3* CA1-1 phenotype. Although we did not observe an apparent effect of the *MRH2* mutation in AF organization, we found that *mrh2-3* root hair growth was more sensitive to Latrunculin B. Moreover, an ARM domain-containing MRH2 fragment could bind to the polymerized actin *in vitro*. Therefore, our genetic analyses, together with cell biological and pharmacological evidence, suggest that the plant-specific kinesin-related protein MRH2 is an important component that controls MT organization and is likely involved in the ROP2 GTPase-controlled coordination of AF and MT during polarized growth of root hairs.

## Introduction

Root hairs are long, thin tubular-shaped outgrowths from root epidermal cells. Root hair development has been conceptually divided into several phases [1]–[3]. The first phase is specification of the hair-producing cells called trichoblasts. Trichoblasts undergo longitudinal growth and are morphologically distinct from non-hair-producing cells called atrichoblasts. The fate specification process is determined by the positional information in alternating cell files. The second phase is root hair growth initiation where trichoblast cell polarity is established, manifested by the bulge formation close to the basal end of the trichoblast. The third phase is tip growth. This phase is characterized by the early-stage slow outgrowth from the bulge to form a swelling of up to about 20–40 µm in length (transition to tip growth) and the subsequent rapid elongation of hairs (tip growth). Significant cellular activities at this phase include extended cytoskeleton (re)organization, rapid exocytosis, nuclear migration toward the tip, and vacuolation. At the end, tip growth arrests, resulting in the fully-grown, mature hairs.

Accumulating evidence show that root hair tip growth is a highly dynamic process, requiring the well-organized cytoskeletons, such as actin filaments (AF) and microtubules (MT), to facilitate active organelle and vesicle transport [2]–[5]. Live cell imaging using green fluorescent protein (GFP) which is fused to the MT markers (such as the MBD domain of mouse MAP4 [6], [7]) or the AF markers (such as the ABD2 domain of *Arabidopsis* FIM1 [8]–[11]) has allowed visualization of MT or AF patterns without causing much inhibitory effect in root hair growth. The critical roles of AF and MT in root hair tip growth have been demonstrated by the alterations of tip growth that are caused by the changes in AF and MT organization. Both drugs and genetic mutations or transgenic modifications have been used to disrupt AF and MT organizations. The drugs include those that disrupt polymerization/deploymerization dynamics of actin (such as Latrunculin B abbreviated here as LatB, and cytochalasin D) and MT (such as Oryzalin abbreviated here as Ory, and taxol) [12]–[15], while the genes mutated or overexpressed include those encoding critical components of actin (such as Actin2, PFN1, FH8 and ADF1) or MT (α–tubulin, MOR1, MRH2) organization [16]–[22]. These studies have led to a general hypothesis: while AF are involved in both tip growth and polarity control, MT are not required for tip growth but important for maintaining the growth direction [1], [3], [13], [14]. Furthermore, AF and MT together form a dynamic network which is important for root hair tip growth. For example, a 30-min pulse application of cytochalasin D broadened the tip of growing hairs, but after the application the tip was quickly recovered to the original growth direction [14]. However, if the root hairs were also treated by Ory (which almost completely disrupted MT organization but did not inhibit hair elongation rates), the growth occurred in a random direction [14]. This result implies that AF can specify the MT direction of cell expansion. However, how the AF and MT interaction is regulated in root hair tip growth remains poorly understood.

Recent findings on the ROP GTPase control of AF and MT organization in pavement cell morphogenesis [23], [24] and in root hair tip growth provide some clues [25]–[27]. ROP, a plant-unique subfamily of Rho family GTPase, is distinct from three other subfamilies in yeast and animals, Rho, Rac and Cdc42, although ROP is sometimes referred to as Rac due to its slightly higher sequence identity with Rac [28], [29]. Accumulating evidence suggest that, as Ras or Rho in yeast and mammals, ROP acts in many aspects of plant growth and development including pollen tube and root hair tip growth [28], [30]–[33]. Transgenic expression of the constitutively active (CA) forms of ROP2, ROP4, ROP6 and ROP11/Rac10 causes proportions of root hairs to become bulbous, a manifestation of the partial disruption of cell polarity [25]–[27]. It was shown that ROPGDI1/SCN1, an GDP/GTP dissociation inhibitor of ROP, facilitates the root hair tip localization for ROP2 which controls, through RHD2, reactive oxygen species production and Ca^2+^ flux in specifying the root hair tip growth [34]–[36]. More interestingly, root hairs in these transgenic plants expressing *CA-rop2*, *rop4*, *rop6, or rop11/rac10* exhibit a net-like arrangement of finer AF and more randomly orientated and shorter MT in the tips for those bulbous hairs, using immunofluorescence [27] or GFP/YFP-mTalin imaging [25], [26]. Furthermore, ROP2 and ROP11/Rac10 are involved in actin cytoskeleton-mediated endocytosis and membrane cycling [25], [37]. However, how ROP GTPases regulate the AF and MT organization in root hair tip growth remains elusive. Studies from pollen tube tip growth and leaf pavement cell morphogenesis suggest a conserved function of Rho family GTPases in yeast, animals and plants, but specific features of the biochemical mechanisms mediated by ROP-regulated AF and MT assembly (such as plant-unique Rop effector proteins called RICs) seem to differ from those of Cdc42/Rac-dependent mechanisms in yeast and animals [23], [30], [38].

To identify the regulatory components involved in the ROP2 control of AF and MT organization during root hair tip growth, we decided to screen for enhancers or suppressors of the CA1-1 transgenic line expressing *CA-rop2* [26]. Here, we report an enhancer mutant (*cae1-1* CA1-1) that showed extreme bulbous root hairs, compared to the mild disruption of root hair tip growth in CA1-1 [26]. *CAE1* encodes a novel allele of the plant-specific, Armadillo (ARM) domain-containing putative kinesin gene *MRH2* [18], and thus *cae1-1* was designated *mrh2-3*. Using GFP-MBD as a reporter for MT, we show that the mutation in *MRH2* causes significant fragmentation and random orientation of MT in both wild-type and CA1-1 backgrounds. Interestingly, although Ory could mimic *mrh2-3* in enhancing the CA1-1 root hair phenotype, the addition of LatB further enhanced the Ory effect. We show that *mrh2-3* root hairs also increased the sensitivity to LatB. Furthermore, an ARM domain-containing MRH2 fragment could bind to the polymerized actin *in vitro*. These results suggest that the plant-specific putative kinesin MRH2 is an important component that controls MT organization and likely acts in the ROP2 GTPase-controlled coordination of AF and MT during polarized growth of root hairs.

## Results

### Isolation and characterization of a *CA-rop2* enhancer involved in root hair tip growth

**Figure 1:**
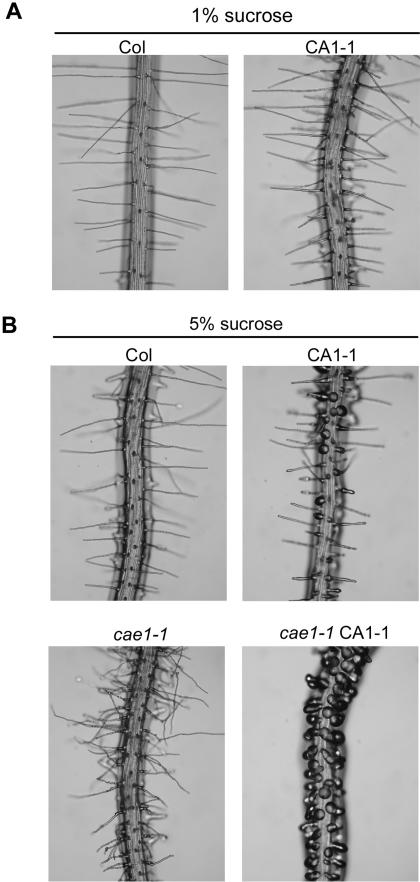
Isolation of a CA1-1 enhancer (*cae1-1*) in root hair tip growth. (A) Col and CA1-1 root hairs grown in the presence of 1% sucrose. (B) Root hairs grown in the presence of 5% sucrose. CA1-1 showed some bulbous root hairs, however all of root hairs in the *CA-rop2* enhancer mutant (*cae1-1* CA1-1) were bulbous. In the Col background, *cae1-1* caused root hairs waving and branching.

To develop a genetic screen for *CA-rop2* enhancers or suppressors, we chose CA1-1, a homozygous transgenic line expressing *CA-rop2* (ROP2^G15V^; described in [39]) and tested the optimal growth conditions for observing its root hair morphologies. Surprisingly, we found that CA1-1 root hair tip growth was very sensitive to sucrose concentrations in the medium. At 1% sucrose, there was virtually no bulbous root hair, but at 5% sucrose, about 30% of root hairs were bulbous (Figure 1). We therefore used the 5% sucrose-supplemented MS medium to screen for both suppressors and enhancers of CA1-1 in root hair tip growth.

A total of 30,000 CA1-1 seeds (M_0_) were mutagenized with ethyl-methane sulfonate (EMS), and M_2_ seeds were harvested from 20 pools of M_1_ plants. Although no suppressor was isolated from our initial screen of 24,000 M_2_ seeds, we found one monogenic, recessive enhancer, designated *cae1-1* CA1-1. As shown in Figure 1B, all of the root hairs in *cae1-1* CA1-1 were bulbous, compared to only a small proportion of the CA1-1 root hairs that were bulbous. This full penetrance phenotype indicates that the loss-of-function mutation on the *CAE1* gene causes *CA-rop2* to completely disrupt root hair tip growth. Interestingly, *cae1-1* (in the Col background) exhibited a wavy and branching root hair phenotype (Figure 1B). Although CA1-1 shows pleiotropic phenotypes [24], [39], we found that *cae1-1* was undistinguishable from Col and did not enhance the CA1-1 phenotypes, with respect to the shapes of cotyledons, leaves and leaf epidermal cells (data not shown). This indicates that *CAE1* is likely specifically involved or at least has a predominant function in root hair tip growth signaling.

### *CAE1* encodes the ARM domain-containing kinesin-related protein MRH2

**Figure 2:**
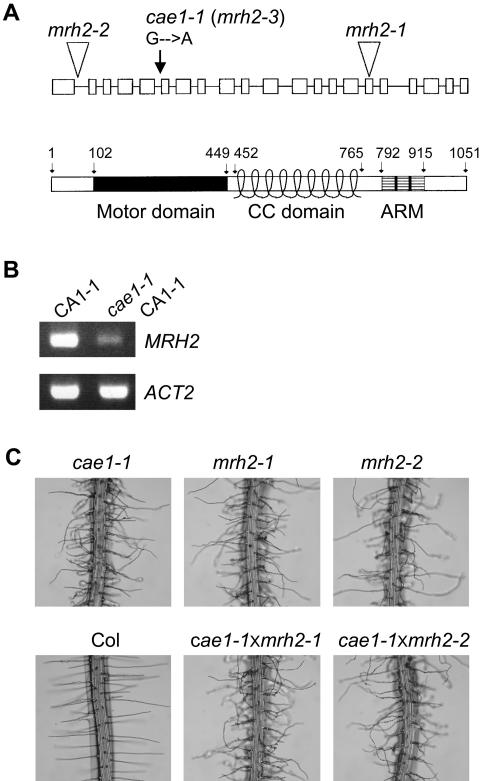
Molecular characterization of the *CAE1*/*MRH2* gene. (A) A schematic representation of the *CAE1/MRH2* genomic structure and domain organization. *Upper panel*, the *CAE1/MRH2* genomic structure. Solid boxes indicate exons and solid lines indicate introns. The *cae1-1* mutation site and the T-DNA insertion sites for *mrh2-1* and *mrh2-2* were indicated. *cae1-1* was renamed to *mrh2-3*. *Lower panel*, domain feature of the 1051 amino acid-containing MRH2 protein. Numbers above indicate the positions of amino acids. Motor domain, the catalytic domain of kinesin motor (102–449); CC, coiled-coil (452–765); ARM, the ARM repeat domain (792–915). (B) RT-PCR analysis of *MRH2* expression in *cae1-1* CA1-1. *ACT2*, the internal control. (C) *cae1-1* failed to complement *mrh2-1* and *mrh2-2*, respectively. Root hairs of F_1_ for both crosses (*cae1-1*×*mrh2-1*, and *cae1-1*×*mrh2-2*) were shown, together with their parents.

To gain molecular insights into the function of the *CAE1* gene product, we used a map-based strategy to clone the *CAE1* gene. *cae1-1* CA1-1 was crossed to L*er* and the resulting F_2_ seedlings were chosen based on the presence of bulbous root hairs and CA1-1-like narrow cotyledons. From a total of 291 F_2_ individuals of *cae1-1* homozygous mutants, we mapped the *CAE1* gene to a 0.5 cM region on chromosome 3. While we were planning to fine map this region, Jones et al. [18] reported that knockout mutants of a putative kinesin gene (At3g54870, designated *MRH2*), which is located within this region, showed similar wavy and branching root hair phenotypes as *cae1-1*. Therefore, we sequenced the *MRH2* genomic DNA and found that there was a G to A mutation that converted a conserved 3′ splicing site AG (at the end of the 5th intron) to AA (Figure 2A, upper panel). RT-PCR analysis indicated that *MRH2* was significantly down-regulated (Figure 2B). Sequencing of seven independently cloned, PCR-amplified *MRH2* cDNA fragments showed that six clones had a 3′splicing site shift by one nucleotide (G, the first base on the 6th exon, constituting a perfect AG splicing site), resulting in frame-shift and subsequent introduction of a stop codon. However, one clone still had correct 3′ splicing at AA, indicating that the 3′splicing site AG is not very well conserved. To confirm that *cae1-1* is allelic to *mrh2*, we crossed two T-DNA knockout lines (*mrh2-1* and *mrh2-2*) respectively into *cae1-1*. Results showed that *cae1-1* failed to complement *mrh2-1* and *mrh2-2* (Figure 2C). This demonstrates that the *CAE1* enhancer mutation is on the *MRH2* gene, and thus *CAE1* was renamed to *MRH2*, and *cae1-1* designated as the *mrh2-3* allele.

*MRH2* was annotated in The Arabidopsis Information Resources (TAIR) website to contain 20 exons, with an ORF of 5,018 bp that encodes 942 amino acids. However, we failed to amplify the *MRH2* cDNA when the antisense primer was designed surrounding the predicted TAA stop codon that was located on the predicted 18th exon (data not shown). We therefore designed primers that spanned from the first to the 20th exons, and the cDNA was successfully amplified. DNA sequencing showed that there was an additional intron (137 bp) spliced out within the TAIR-annotated 18th exon. This splicing eliminated the TAIR-predicted TAA stop codon, and therefore the TAIR-predicted 3′ UTR for the final two exons become the coding regions. The correct genomic structure for the ORF is shown in Figure 2A (upper panel). As a result, *MRH2* has an ORF of 5,819 bp, with the CDS (translated region from ATG to TGA stop codon) of 3,156 bp.

Based on the above experimentally verified genomic annotation, *MRH2* encodes a plant-unique kinesin with 1051 amino acids. The putative kinesin MRH2 contains a conserved motor domain (amino acids 102–449) close to the N-terminus, followed by nine coiled-coil (CC) domains in the middle, and three Armadillo/β-catenin-like (ARM) repeats (amino acids 792–915) close to the C-terminus (Figure 2A, lower panel). A phylogenetic analysis suggests that MRH2 is an atypical KHC (kinesin heavy chain) lacking a light-chain-binding domain [40]. KHC belongs to Kinesin-1 family kinesins that can transport vesicles or organelles along with MT, by forming a tetramer with itself and kinesin light chain molecules. However, a more detailed phylogenetic analysis using several methods strongly indicates that it is a plant-unique kinesin that does not belong to any of 14 known kinesin families [41]. The ARM repeats, each with approximately 40–45 amino acids, are only present in MRH2 and two other MRH2-related plant kinesins (encoded by At1g01950 and At1g12430, respectively) in *Arabidopsis* and one non-plant (leishmania) kinesin [41]. The ARM domain is believed to be involved in protein-protein interactions [42] and may be critical for the cellular function of the putative kinesin MRH2.

### *MRH2* promoter is active throughout root hair development

**Figure 3:**
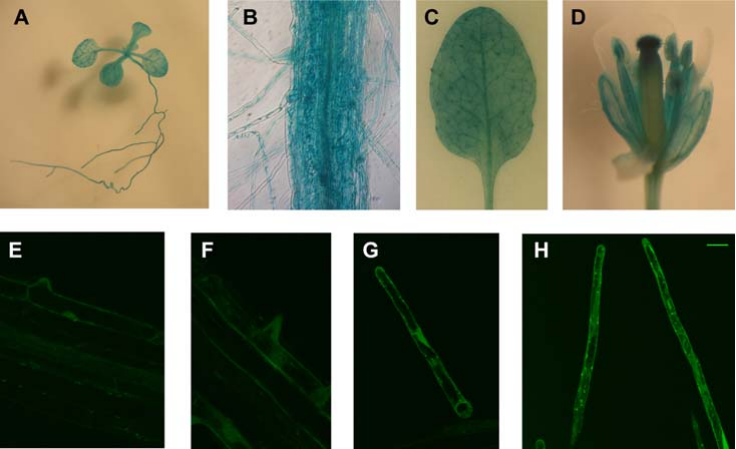
*MRH2* promoter activity in root hairs. Tissue/cell type expression patterns were revealed by histochemical examination of the 955 bp *MRH2* promoter activity using the GUS (A–D) or GFP (E–G) reporters. Shown are a 2-week-old young seedling (A), part of the root with root hairs (B), a leaf (C) in which trichomes were strongly stained but epidermal cells were weakly stained, and a flower (D). Root hairs at various stages (E–H) were also shown. The bar in (H) represents 20 µm in (E–H).

To gain further understanding of MRH2 function in root hair tip growth, we studied its promoter activity by using transgenic lines expressing the β-glucuronidase (*GUS*) or *GFP* reporters driven by the 955 bp fragment (immediately upstream of the ATG codon) of the *MRH2* promoter. Several homozygous lines were used for the observation. The GUS staining pattern indicates that *MRH2* is actively expressed in all parts of the young seedling including cotyledons, true leaves, and the root (Figure 3, A and B). Surprisingly, the *MRH2* promoter was very active in vascular tissues of leaves but only weakly in leaf epidermal cells (Figure 3C). It was also strongly activated in trichomes (Figure 3C), although trichome morphogenesis was not affected by the *MRH2* mutation (data not shown). The promoter is also active in the floral organs except in petals (Figure 3D). However, we did not see the trichoblast-specific activity of the *MRH2* promoter as it was active in both trichoblasts and atrichoblasts in the roots (Figure 3B). Using GFP as a reporter, the *MRH2* promoter was revealed to be active from root hair initiation and tip growth phases to growth arrest (Figure 3, E–H).

### The *MRH2* mutation alters cortical MT organization

Jones et al. [18] cited as unpublished results that MT distribution in the *mrh2* knockout mutants exhibited an unusual pattern, but no detail was provided. Therefore, we decided to characterize the patterns of cortical MT organization in *mrh2-3*, CA1-1 and *mrh2-3* CA1-1. GFP-MBD was first transformed into Col and *mrh2-3*, respectively, and then the resulting transgenic plants were respectively crossed to CA1-1 and *mrh2-3* CA1-1. The resulting transgenic plants and the F_1_ plants that contain GFP-MBD were used for visualizing cortical MT organization in these four genotypes under fluorescent confocal microscope.

**Figure 4:**
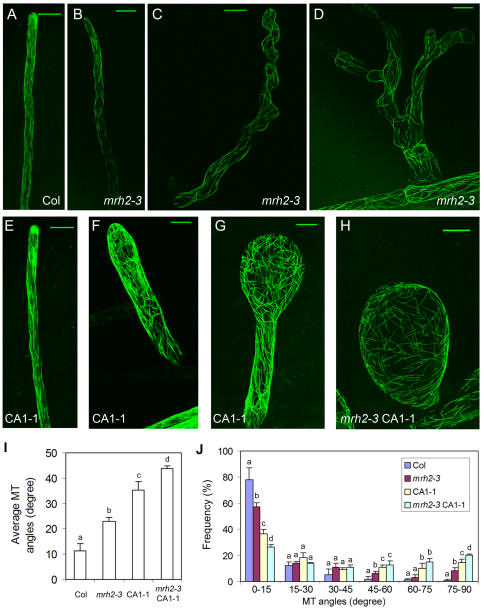
MT organization in *mrh2-3*, CA1-1, and *mrh2-3* CA1-1 root hairs. Representative GFP-MBD images in various genotypes that contain the 35S:GFP-MBD construct. Images were projections of 20–50 confocal sections, separated by 1 µm distance. The bar represents 20 µm. (A) Col. (B–D) MT images from three typical root hairs of *mrh2-3*: slight waving (B), severe waving (C), and severe waving and branching (D). (E–G) MT images from root hairs showing three representative types of CA1-1 root hairs: normal hair (E), swelling hair (F) and hair with bulbous tip (G). (H) Bulbous root hair of *mrh2-3* CA1-1. (I–J) Quantitative analysis of MT orientation. Angles of MT relative to the root hair growth axis were measured. (I) The average MT angles for each root hair were calculated to show the overall MT angles for single root hair. (J) Distribution of MT angles. The data represents the average of three replicates, each with a total of about 180 MTs from 3–7 root hairs. Different letters above the column indicate a statistical significant difference (p<0.05) between genotypes within the same category.

Live cell imaging of GFP-MBD showed that cortical MT in *mrh2-3* exhibited fragmentation and random orientation (Figure 4, B–D), as compared to longitudinal MT cables observed in Col (Figure 4A). This pattern seemed to be correlated with the severity of root hair phenotype in *mrh2-3* because the more severe waving and/or branching root hairs had more obvious fragmentation and random orientation of MT (Figure 4, B–D). Similar observations were made in CA1-1 root hairs: the normal root hairs of CA1-1 did not seem to dramatically change the orientation of MT (Figure 4E), while the swelling hairs (Figure 4F) and, in particular, the root hairs with the bulbous tips (Figure 4G), showed dramatic fragmentation and random orientation of MT. In *mrh2-3* CA1-1, all of root hairs were bulbous and showed the most extensive MT fragmentation and random orientation (Figure 4H). The quantification of the MT angles relative to the root hair growth axis showed that, on average, the angles for each root hair increased in the order of Col, *mrh2-3*, CA1-1, and *mrh2-3* CA1-1 (Figure 4I). The distribution of MT with various angles confirmed that *mrh2-3* and CA1-1 had less MT in the range of 0–15 degrees and more MT in the range of 45–60, 60–75 or 75–90 degrees (Figure 4J). As expected, *mrh2-3* CA1-1 root hairs had the largest proportion of MT with the angles in the range of 75–90 degrees, and in this range the MT angles decreased in the order of Col, *mrh2-3*, CA1-1, and *mrh2-3* CA1-1. In total, about 50% MT in *mrh2-3* CA1-1 root hairs were in the range of 45–90 degrees, while Col, *mrh2-3* and CA1-1 had only approximately 5%, 15%, and 36% respectively (Figure 4J). Taken together, these results show that the kinesin-related protein MRH2 is important for maintaining MT orientation and stabilizing MT.

**Figure 5:**
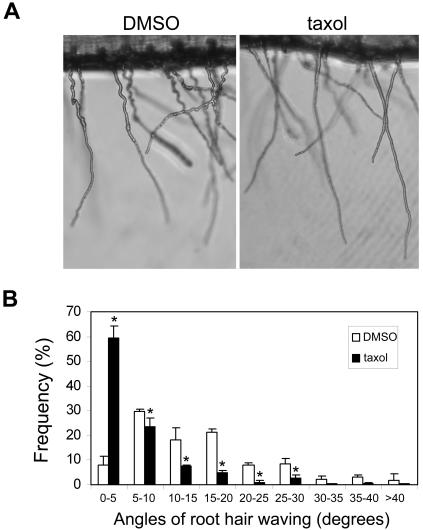
Taxol reduced *mrh2-3* root hair waviness. Three-day old seedlings were transferred to the 1 µM taxol-containing medium for two days of further growth. Representative root hairs were shown in (A), and the distribution of the waving angles of root hairs were presented in (B). The angles for each waiving relative to the root hair growth axis were measured. The bar represents the average and the SD of three replicates. Each replicate has 3 or 4 roots, with a total of approximately 30 root hairs. The asterisk (*) above the column indicates a significant difference (p<0.05) compared to the DMSO control within the same category.

To confirm that the *MRH2* mutation-caused MT instability is important for the control of root hair growth orientation, taxol, another drug known to stabilize MT, was applied to *mrh2* root hairs. As shown in Figure 5, 1 µM taxol reduced root hair waving in *mrh2-3*. Although the taxol treatment did not completely rescue the *mrh2-3* root hair waving phenotype, it dramatically decreased the waving angles of root hairs relative to the root hair growth axis, compared to the DMSO control (Figure 5B). For example, in the DMSO control, the majority (70%) of waving angles were in the range of 5–20 degrees, while in the taxol treatment about 60% of the waving angles were less than 5 degrees. This result supports that MRH2 has an important role in stabilizing MT during root hair directional growth.

### Treatment of Ory, or its combination with LatB, in CA1-1 mimics the effects of the *MRH2* mutation

**Figure 6:**
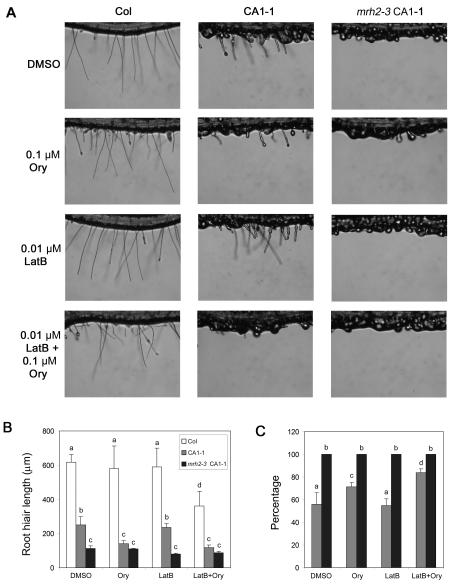
Combination of LatB and Ory treatments in CA1-1 mimics the enhancer phenotype. Seedlings for all of the four genotypes were treated identically as in Figure 7. Representative images are shown in (A), and quantitative analyses of root hairs are shown in (B) and (C). For each genotype/treatment combination, a total of 4 or 5 roots (each with about 20 root hairs on the same root region) were used in the experiment. The average and the SD of the root hair lengths (B) and of the percentage of very short root hairs showing swelling or bulbous shapes (C) are shown. Note that (B) and (C) have the same column labeling, and in (C) there are no very short, swelling or bulbous root hairs. Different letters above the column indicate a statistical significant difference (p<0.05).

If the *MRH2* mutation-caused disruption of MT organization is responsible for enhancing the *CA-rop2* phenotype, we would expect that treatment of Ory in CA1-1 would phenocopy *mrh2-3* CA1-1. Seedlings of Col, CA1-1 and *mrh2-3* CA1-1 were treated by the low concentration (0.1 µM) of Ory which did not affect root hair lengths in Col (Figure 6) but greatly disrupted cortical MT organization in root hairs (data not shown). As expected, we found that Ory suppressed root hair growth in CA1-1 to the length similar to that in *mrh2-3* CA1-1 (Figure 6B). However, when the root hair morphology was assessed, Ory increased the percentage of very short hairs showing swelling or bulbous shapes in CA1-1 to 71% only, compared to 56% for the DMSO control and 100% for *mrh2-3* CA1-1 regardless of the treatment (Figure 6C). This indicates that Ory could enhance the *CA-rop2* root hair tip growth defect but was not sufficient to convert all of CA1-1 root hairs to bulbous ones as in *mrh2-3* CA1-1.

We then tested the possibility that AF might be involved in *mrh2-3* CA1-1 root hair tip growth, given that AF and MT cross-talk is important for tip growth [14] and that CA-rop2 alters MT (Figure 4, F and G) and AF organization [26]. However, unlike Ory, low concentration (0.01 µM) of LatB did not suppress root hair growth (Figure 6, A and B), nor increased the percentage of very short hairs showing swelling or bulbous shapes in CA1-1 (Figure 6C). Interestingly, the combination of LatB and Ory further increased the percentage of very short hairs to 84% (Figure 6C), compared to Ory alone. This combinatory effect of Ory and LatB on root hair growth was also observed in Col, although there was no such short hair exhibiting swelling or bulbous shapes observed in Col (Figure 6). These results indicate that the *MRH2* mutation might also affect the AF organization or the cross-talk between MT and AF during root hair tip growth.

### The *MRH2* mutation enhances the sensitivity to LatB in root hair growth

**Figure 7:**
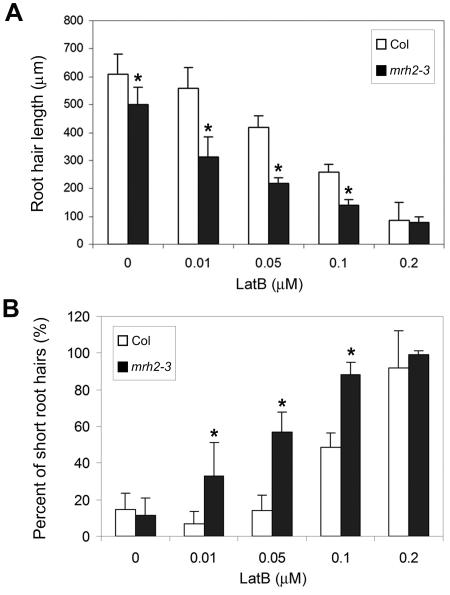
Hypersensitivity of *mrh2-3* root hairs to LatB. Three-day-old seedlings that were grown in the drug-free medium and then transferred to the LatB-containing medium for two additional days. The solvent DMSO was used as the 0 µM control. Col, the wild-type. (A) Quantitative analysis of *mrh2-3* root hair length in response to LatB. A total of 12–15 root hairs for each root (except about five root hairs for 0.2 µM LatB treatment) were measured, and the average and the SD of eight roots are shown. (B) Proportions of short root hairs in *mrh2-3* after LatB treatments. The same data as in (A) were analyzed. Root hairs shorter than 220 µm were classified as short root hairs. The asterisk (*) above the column indicates a significant difference (p<0.05) compared to Col under the same treatment.

To investigate the possibility that the *MRH2* mutation affects AF organization or dynamics, we tested *mrh2-3* root hair growth response in the presence of various doses of LatB for two days. Although LatB at a low concentration (0.01 µM) did not affect root hair length in Col, it suppressed *mrh2-3* root hair growth by approximately 40% (Figure 7A). At higher concentrations (0.05 and 0.1 µM) of LatB, root hair elongation in Col was suppressed, but *mrh2-3* root hairs showed stronger suppression. At 0.2 µM, Col and *mrh2-3* root hairs were dramatically suppressed and had similar lengths. The frequencies of short root hairs (shorter than 220 µm) exhibited a similar pattern (Figure 7B). To determine whether the *MRH2* mutation alters pattern of AF organization, a GFP reporter line for visualizing cortical AF, *35S:ABD2-GFP* [11], was respectively crossed to *mrh2-3*, CA1-1 and *mrh2-3* CA1-1. However, we did not observe any dramatic difference in AF organization between *mrh2-3* and Col, or between CA1-1 and *mrh2-3*/CA1-1 (Figure S1), although as reported by others [25]–[27], we also observed the formation of extensive AF networks and the extension of thick actin cables to the apex in CA1-1, which was not usually found in Col [25]–[27]. It is possible that the changes in AF organization caused by the *MRH2* mutation were too subtle to be revealed by the ABD2-GFP reporter.

### The C-terminal ARM domain-containing MRH2^649-1051^ fragment binds to the polymerized actin *in vitro*

**Figure 8:**
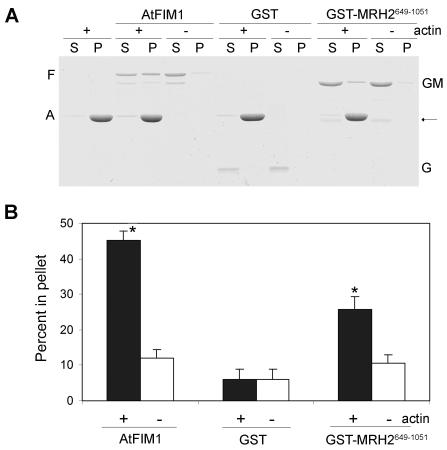
Binding of the ARM domain-containing MRH2^649-1051^ polypeptide to the polymerized actin *in vitro*. (A) A high-speed co-sedimentation assay for the binding of GST-MRH2^649-1051^ Fragment to the polymerized actin. Equal amounts of GST-MRH2^649-1051^ proteins were loaded in the presence (+) or the absence (−) of the polymerized actin. After high-speed centrifugation, proteins from the supernatant (S) and the pellet (P) were separated on the SDS-PAGE gel. AtFIM1 was used as a positive control, while GST as a negative control. The position of actin (A) and AtFIM1 (F) are marked at the left, and the positions of GST (G) and GST-MRH2^649-1051^ (GM) are indicated at the right. Note that GST-MRH2^649-1051^ might have a slight degradation (bands indicated by the arrow on the right). (B) Quantitative analysis of the percentage of proteins in the pellet. The three resulting gels were scanned to determine the amount of proteins that was present in the supernatant and the pellet fractions. After the background subtraction, the percentage of proteins that co-sedimented with the polymerized actin was calculated by dividing the intensities in the pellet from the sum of that in the pellet and the supernatant. The bar represents the average and the SD of three repeated experiments. The asterisk (*) above the column indicates a significant difference (p<0.05) compared to the control of no actin.

To test whether MRH2 can directly bind to AF *in vitro*, we initially attempted to perform a co-sedimentation assay using the fusion protein of GST with the full-length MRH2. However, we failed to purify GST-MRH2 in *E. coli*. Therefore, we decided to express and purify a C-terminal 403 amino acid fragment (MRH2^649-1051^) that contains part of the coiled-coil region and the whole ARM domain (Figure 2A, lower panel). GST-MRH2^649-1051^ could be purified, and therefore we tested its direct binding with the polymerized actin using a high-speed co-sedimentation assay. AtFIM1, a well-characterized actin cross-linking protein [43], was used as a positive control for comparison of the binding efficiency. As shown in Figure 8A, the majority of the polymerized actin sedimented at 200,000 g. In the absence of the polymerized actin, only a small proportion of GST-MRH2^649-1051^ or AtFIM1 sedimented. However, more of the GST-MRH2^649-1051^ proteins co-sedimented in the presence of the polymerized actin, although the proportion of GST-MRH2^649-1051^ in the pellet was smaller than that of AtFIM1. The binding of GST-MRH2^649-1051^ to the polymerized actin was unlikely due to GST, as rarely detectable GST sedimented in both the absence and the presence of the polymerized actin (Figure 8A). Quantitative analysis (Figure 8B) showed that, while 10.5% of GST-MRH2^649-1051^ was present in the pellet in the absence of the polymerized actin, 25.8% of GST-MRH2^649-1051 ^sedimented in the presence of the polymerized actin, indicating that approximately 15% of MRH2^649-1051^ likely binds to the polymerized actin. However, the affinity of MRH2^649-1051^ to the polymerized actin was about 50% lower than that of AtFIM1. This result indicates that the ARM domain-containing C-terminus of MRH2 can, to some extent, bind to the polymerized actin *in vitro*.

### The N-terminal motor domain-containing MRH2^1-449 ^fragment binds to the polymerized tubulin *in vitro*

**Figure 9:**
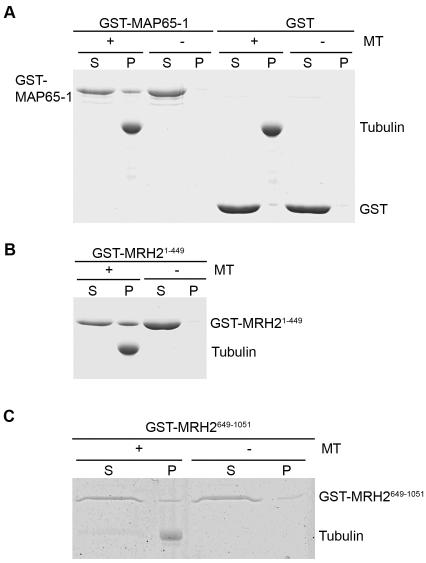
Co-sedimentation of MRH2^1-449^ and MRH2^649-1051^ with the polymerized tubulin. Equal amounts of proteins were loaded in the presence (+) or the absence (−) of the polymerized tubulin (MT). After high-speed centrifugation, proteins from the supernatant (S) and the pellet (P) were separated on the SDS-PAGE gel. (A) Control experiments for the co-sedimentation assay. GST-MAP65-1 (5 µM, positive control) or GST (10 µM, negative control) were loaded to 5 µM MT. (B) GST-MRH2^1-449^ (10 µM) was loaded in the presence or absence of 5 µM MT. (C) GST-MRH2^649-1051^ (1 µM) was loaded in the presence (+) or the absence (−) of 2 µM MT.

To determine whether the kinesin-related MRH2 protein can bind to MT, a co-sedimentation assay with the polymerized tubulin was similarly performed. We observed that GST-MRH2^1-449^ which contains the motor domain (Figure 2A, lower panel) could bind to MT as strongly as a MT-associated protein, MAP65-1 (Figure 9, A and B). The strong binding to MT for these GST fusion proteins was unlikely due to GST, as the presence or absence of MT resulted in a similarly very small proportion of GST in the pellet (Figure 9A). Therefore, this result indicates that MRH2 is likely a functional kinesin. In contrast, GST-MRH2^649-1051 ^did not co-sediment with MT, like GST alone (Figure 9C). Therefore, the lack of binding to MT for GST-MRH2^649-1051 ^suggests that its binding to actin (Figure 8) is less likely the result of non-specific aggregation with the polymerized actin.

## Discussion

### The putative kinesin MRH2 has a predominant role in polarized growth of root hairs

Kinesins belong to a group of cytoskeletal motor proteins that are critical for mitosis, meiosis, and transport of various organelles and vesicles along MT [40]. In *Arabidopsis*, there are a total of 61 kinesins, and most of these kinesins can be grouped into some of 14 subfamilies of the eukaryotic kinesin superfamily [40], [41], [44]. However, being grouped into certain subfamilies does not always indicate identical or similar functions [44]. Therefore, it remains a significant challenge to reveal the functions for all of 61 *Arabidopsis* kinesins. Genetic studies have provided some clues to the diverse functions of some kinesins in plant growth and development, but only several of these exhibit morphological phenotypes, such as trichome branching, cellulose microfibril orientation, root hair waviness and branching, and overall plant growth and organ size (such as in [45]–[50] and reviewed in [44]).

MRH2 is one of the three putative kinesins that contain the ARM domain in *Arabidopsis* and they together have been placed as a plant-unique kinesin subfamily [41]. MRH2 was recently shown to be involved in the control of root hair initiation and growth direction [18]. However, given by the subtle phenotype of the *MRH2* T-DNA knockout lines, it would be difficult to envision its function in the ROP2-controlled root hair tip growth as reported here. Through an enhancer screen, we have provided further evidence that MRH2 has a predominant role in the control of root hair growth. The *CA-rop2* enhancer is a novel loss-of-function allele of *MRH2* (*cae1-1*/*mrh2-3*) and in the Col background it shows the same root hair waving and branching phenotype (Figure 2) as two other alleles [18]. Consistent with its function in root hair tip growth, we show that the *MRH2* promoter is active throughout root hair development. Importantly, although the promoter is also active in leaf trichomes and pavement cells, the *MRH2* mutation does not alter trichome and pavement cell morphogenesis in both Col and *CA-rop2* plants (data not shown). In addition, we have found that a T-DNA knockout mutant (Salk_124908) for one (encoded by At1g01950) of the two *MRH2*-closely related, ARM domain-containing kinesins does not exhibit any root hair growth defect (data not shown).

### A role for MRH2 in coordinating MT and AF during root hair tip growth

Our observations that the *MRH2* mutation causes cortical MT to be randomly oriented and fragmented and greatly enhances the *CA-rop2*-activated disruption of MT organization (Figure 4) suggest that MRH2 is important for stabilizing MT or maintaining MT orientation. This role is further supported by the drug studies in which the MT stabilization drug, taxol, can partially rescue the wavy root hair phenotype of *mrh2-3* (Figure 5) and the MT depolymerization drug, Ory, can mimic the effect of *mrh2-3* in enhancing the CA1-1 root hair tip growth effect (Figure 6). In addition, the N-terminus of MRH2 (MRH2^1-449^) which contains the motor domain can bind to the polymerized tubulin *in vitro* (Figure 9). Taken together, these results show that MRH2 is important for stabilizing cortical MT or maintaining normally longitudinal orientation of MT during root hair tip growth. However, the mechanism by which MRH2 regulates MT organization remains unknown. MRH2 contains the ARM domain which is believed to be involved in protein-protein interactions [40], [42], raising the possibility that this domain might facilitate the MRH2 function in MT organization or transport. Interestingly, the ARM domain absent in yeast and animal kinesins has been found to be present in the Kinesin-2 family-associated protein KAP3 in animals [40], [41]. One hypothesis proposed is that MRH2 or its related kinesins might function as Kinesin-2 family kinesins which do not exist in higher plants [40]. In this hypothesis, the ARM domain of MRH2 might interact with an unknown regulatory protein involved in MT organization or an unknown cargo-binding protein involved in vesicle/organelle transport.

Unexpectedly, we have found that MRH2 is possibly involved in the coordination of MT and AF organization or dynamics in root hairs. Results from the drug experiments indicate a possible role for MRH2 in the control of AF organization or dynamics or the coordination of AF and MT. First, *mrh2* root hairs were more sensitive than wild-type to LatB (Figure 7). Second, the addition of LatB enhanced the Ory effect in mimicking the effect of the *MRH2* mutation in CA1-1 (Figure 6). Although we did not observe any dramatic alterations in AF organization in *mrh2-3*, or in *mrh2-3* CA1-1 compared to CA1-1 (Figure S1), this might be due to either the subtle changes in AF organization which could not be revealed by the ABD2-GFP marker, or the transient or dynamic AF changes which need to be studied further. The possibility that the increased sensitivity to LatB might be secondary effect can not be excluded, although LatB is a relatively specific actin depolymerization drug which acts by forming a high affinity complex with monomeric actin. Interestingly, we have observed that the ARM domain-containing MRH2 fragment (MRH2^649-1051^) could bind to the polymerized actin in an *in vitro* high-speed co-sedimentation assay (Figure 8), although its affinity was 50% lower than that in AtFIM1 [43]. The binding of this truncated MRH2 fragment to actin is less likely the result of non-specific aggregation with the polymerized actin, because it did not seem to co-sediment with the polymerized tubulin (Figure 9). This finding is a surprise to us, as MRH2 does not contain any known actin binding domain. It has been reported that several *Arabidopsis* kinesins contain the actin-binding CH domain [41], [44], [51], as in the case of a CH domain-containing cotton kinesin (GhKCH1) which is shown to bind to the polymerized actin *in vitro* [51]. However, none of these *Arabidopsis* kinesins has been demonstrated to bind to the polymerized actin *in vitro*. Indeed, it remains unknown if any of the 61 members of *Arabidopsis* kinesins is directly involved in the control of AF organization. Therefore, one of the future directions is to confirm the MRH2-actin binding *in vivo* and determine its functional significance.

The mechanism by which MRH2 might coordinate MT and AF organization remains unclear. Studies using MT and AF drugs suggest that AF and MT together form a dynamic network during root hair tip growth: on the one hand, MT are important for maintaining the growth direction, and alterations in MT lead to changes in AF organization; on the other hand, AF are involved in both tip growth and polarity control, and AF can specify the MT direction of cell expansion [1], [3], [13], [14]. Although how MRH2 coordinates AF and MT organization or dynamics remains to be determined, MT organization is the basis for MRH2 involvement in the MT and AF cross-talk during root hair tip growth. This is because LatB alone had no effect in the CA1-1 root hair phenotype, but it could further stimulate the Ory enhancement effect in CA1-1 (Figure 6). This type of indirect interaction between kinesin and actin has been reported in the yeast tip growth model. In this model, Tea2p, a kinesin-like motor protein, redirects actin assembly through the “polarisome” complex that interacts with a formin called For3p [52]. Interestingly, some of the yeast “polarisome” components have homologs in *Arabidopsis* [3], [53], and therefore, MRH2 might act through a similar mechanism for its cross-talk with AF during root hair tip growth. However, the possibility for a direct interaction between MRH2 and AF can not be excluded, as we have shown the *in vitro* interaction between MRH2^649-1051^ and the polymerized actin (Figure 8). The presence of the ARM domain might facilitate the direct binding of MRH2 to AF, but whether such binding occurs *in vivo* remains to be determined.

### Hypothetical models for the ROP2-MRH2 functional interaction in the control of MT and AF cross-talk

Our genetic evidence provides a functional link between ROP2 GTPase and the putative kinesin MRH2 in the control of MT and AF cross-talk during root hair tip growth, but the mechanism of their functional interaction remains unknown. We have observed that the combination of LatB and Ory treatments in CA1-1, but not in *mrh2-3*, could mimic the *mrh2-3* CA1-1 phenotype (Figure 6 and data not shown). This suggests that regulation of the ROP2 GTPase activity is essential for the control of root hair tip growth. We further show that inactivation of MRH2 alone and constitutive activation of ROP2 alone could similarly disrupt MT organization and their combination completely disrupted MT organization and root hair tip growth (Figure 4). There exist at least two possibilities for the functional interaction between MRH2 and ROP2. One possibility is that MRH2 might act as a common, negative component of ROP2 signaling in the control of MT organization and MT-AF cross-talk. In this case, ROP2 might regulate MT organization through MRH2. It has been demonstrated in leaf epidermal cell morphogenesis that localized ROP2 activation can interact with and suppress its effector protein RIC1 which is required for the formation of MT bundles at neck regions [23]. Interestingly, the RIC1-mediated MT organization in turn suppresses ROP2 activation in the indentation zone, leading to the suppression of RIC4 which promotes AF formation in the regions of growing lobes and the ultimate determination of leaf epidermal cell shape [23]. If similar ROP-RIC signaling pathways operate in root hair tip growth, MRH2 might be regulated by certain member of RICs, resulting in the control of MT organization. Consequently, the MRH2-mediated MT organization then feedback regulates AF organization, or MRH2 might directly bind to AF to coordinate AF and MT. Alternatively, the MRH2-controlled MT organization might affect the ROP2 GTPase signaling in the regulation of MT and AF organization through RICs. Therefore, testing the role of RICs in root hair tip growth and isolating the MRH2 protein complex in the future will help to address the essential question regarding how MRH2 interacts with the ROP2 GTPase pathway in the control of MT and AF cross-talk in the polarized growth of root hairs.

## Materials and Methods

### Plant materials and growth conditions

*Arabidopsis thaliana* Columbia (Col), transgenic plants and mutants were used in this study. For seedling growth on the plates, seeds were sterilized and placed on 1% PhytoBlend-solidified half-strength MS medium with 1–5% sucrose, and cold-treated at 4°C for 2–4 days. After germination, seedlings were then grown horizontally or vertically on the plate in the growth chamber at 22°C with 16-hour light and 8-hour dark.

### Enhancer isolation, gene cloning and complementation test

A total of 30,000 CA1-1 seeds that are homozygous for the *CA-rop2* transgene [39] were mutagenized using 0.3% ethyl-methane sulfonate (EMS) and then grown into 20 pools of M_1_ plants. About 24,000 M_2_ seeds were initially screened for *CA-rop2* enhancers by vertically growing on the 5% sucrose-supplemented half-strength MS medium for 5–10 days. Seedlings that showed all of bulbous root hairs were isolated as putative enhancer mutants. The putative mutants were back-crossed to Col twice before being used for genetic analysis.

Molecular cloning of the *cae1* mutant gene followed the map-based cloning strategy [54]. In brief, the resulting *cae1-1* mutant in the CA1-1 background (*cae1-1* CA1) was crossed to the L*er* ecotype and the F_2_ seedlings showing both bulbous hairs and cotyledon shape identical to CA1-1 were used for genotyping. Based on the marker genotyping results from a total of 291 plants, the *CAE1* mutant gene was mapped to a region between the two markers on the BAC clones on chromosome 3: Marker 470805 on T5N23 (using PCR primers T5N23M1S and T5N23M1A) and Marker 476269 on T22E16 (primers T22E16M1S and T22E16M1A). Primer sequences were available in Table S1. Since Jones et al. [18] reported that knockout mutants of a putative kinesin gene designated *MRH2* (At3g54870) which is located within this region showed similar wavy and branching root hair phenotypes as *cae1-1*. We therefore PCR amplified and sequenced the *MRH2* open reading frame.

Genetic complementation tests were performed by crossing *cae1-1* in the Col background to *mrh2-1* and *mrh2-2*, respectively. These two T-DNA knockout lines were directly ordered from the ABRC at Ohio State University and had been described elsewhere [18].

### Reverse transcriptase-mediated PCR

Total RNA was extracted with TRIzol (Invitrogen, USA) from seven-day-old seedlings, and 5 µg RNA were reverse transcribed in a 20-µL reaction using Superscript III reverse transcriptase and Oligo(dT)_12-18_ primer (Invitrogen), according to the instructions provided by the venders. PCR analysis was performed using the Taq DNA polymerase (GenScript, USA), with gene specific primers and *ACT2* as the internal control according to Xin et al. [55]. The *MRH2* full length cDNA was amplified using the sense primer YZP77 (with a *Bam*H I site incorporated) and the antisense primer YZP79RR (with an *Xba* I site incorporated; Table S1). For determination of *MRH2* expression, an internal 702 bp cDNA fragment was amplified using the gene specific primers: the sense primer MRH2-3S, and the antisense primer MRH2-4A (Table S1).

### Construction of GFP-MBD fusion protein

35S:GFP-MBD was first constructed by PCR using the mouse total cDNA kindly provided by Dr. Z. Chen, essentially by following the strategy published by Marc et al. [6]. Two primers were used: ZZP71 (sense, incorporating a *Bgl* II site), and ZZP72 (antisense, incorporating an *Xba* I site; Table S1). The amplified cDNA fragment was digested by *Bgl* II and *Xba* I, and then cloned into the pCAMBIA3301-based binary vector GZ0 that contains GFP and the CaMV 35S promoter and terminator, giving rise to the 35S:GFP-MBD vector. The construct was first introduced into *Agrobacterium tumefaciens* strain GV3101 and the floral dip method [56] was used to transform *Arabidopsis* plants.

### Fluorescent confocal microscopy

AF and MT were visualized under a Bio-Rad Radiance 2000 confocal laser scanning device (Carl Zeiss Microimaging, Inc., NY, USA). To visualize AF in different backgrounds, they were crossed to the *35S:ABD2-GFP* transgenic line [11] kindly provided by Dr. Elison B. Blancaflor, and the resulting progeny that contain ABD2-GFP and show expected root hair phenotypes were used for AF organization observation. To visualize MT in different backgrounds, *35S:GFP-MBD* was transformed into Col and *mrh2-3*, respectively. The resulting transgenic plants were then respectively crossed to CA1-1 and *mrh2-3* CA1-1 backgrounds. The F_1_ or T_2_ seeds were used for AF visualization under fluorescent confocal microscope. Single sections at a 1 µm distance were collected and the projections were made. These images were then analyzed using the NIH ImageJ 1.37 software (website http://rsb.info.nih.gov/ij/) and processed using Photoshop.

### Drug treatments

LatB, Ory and taxol were purchased from Aldrich-Sigma (St. Louis, MO, USA). They were dissolved as 1 mM stock using DMSO. LatB, Ory and taxol at the specified final concentrations were prepared in the half-strength MS medium. Seedlings were first grown on the drug-free medium for 3 days after 2–4 days of cold treatment. They were then transferred to the drug-containing medium for additional one or two days. The effects of LatB, Ory and taxol on root hair morphology were observed under light microscope.

### Construction of promoter::GUS or GFP reporter lines and GUS assays

For the *MRH2* promoter::*GUS* construct, a 955 bp promoter fragment immediately upstream of ATG was PCR amplified from genomic DNA using the high fidelity DNA polymerase Elongase® (Invitrogen, USA) and the gene-specific primers (YZP74 and YZP76; Table S1) with the underlying bases indicating the introduced restriction enzyme sites, *Eco*R I and *Nco* I, respectively. The amplified DNA fragment was then cloned into the *Eco*R I and *Nco* I sites of the binary vector pCAMBIA1301 (B4) that contains GUS and the CaMV35S terminator, giving rise to the CAE1P vector, YZ54. The promoter fragment was also cloned into the GFP-containing vector, GZ0, to replace the CaMV 35S promoter, resulting in YZ55. Homozygous transgenic lines that contain YZ54 and YZ55, respectively, with a single T-DNA insertion were obtained by hygromycin selection. Histochemical GUS activity assays were performed on homozygous transgenic seedlings as described [57].

### *In vitro* binding of the C or N-terminal MRH2 polypeptides to the polymerized actin or tubulin

GST-MRH2^1-449^ and GST-MRH2^649-1051^ were constructed by cloning the PCR amplified cDNA fragments encoding amino acids 1–449 and 649–1051 of MRH2 into pGEX-KG. The primers YZP105 and YZP79RR with the *Bam*H I and *Xba* I sites incorporated respectively (Table S1), were used to amplify MRH2^649-1051^, and the resulting GST-MRH2^649-1051^ vector was designed YZ105. For GST-MRH2^1-449^ (designated YZ112), the primers YZP114 and YZP115, with the underlying restriction sites, *Xba* I and *Xho* I, incorporated respectively (Table S1), were used to amplify the fragment MRH2^1-449^. GST, GST-MRH2^649-1051^ and GST-MRH2^1-449^ were expressed in the *E. coli* strain BL21 (DE3). After sonication in the protease inhibitors-containing buffer [58], the crude was clarified by centrifugation and the supernatant was loaded onto the glutathione-Sepharose column. After elution with 20 mM glutathione, the fusion protein was dialyzed against Buffer G (2 mM Tris-HCl, pH 8.0, 0.01% NaN3, 0.2 mM CaCl2, 0.2 mM ATP, and 0.2 mM DTT), aliquoted and stored at −80°C. Protein concentration was determined by Bradford assay (Bio-Rad, Hercules, CA, USA).

For *in vitro* binding of GST-MRH2^649-1051^ to actin, a high-speed co-sedimentation assay as described [43], [59] was used to examine its the binding to the polymerized actin. AtFIM1 was used as a positive control and the purification of GST-AtFIM1 was described elsewhere[43]. Actin was purified from rabbit skeletal muscle acetone powder as described [60], and monomeric Ca-ATP-actin was purified by Sephacryl S-300 chromatography in G buffer (5 mM Tris-HCl, pH 8, 0.2 mM ATP, 0.1 mM CaCl_2_, 0.5 mM DTT, and 0.1 mM azide), according to Pollard [61]. Actin concentration was determined by spectrometry (A290 of 0.63 being equivalent to 1 mg/mL). The procedure was briefly described here. All proteins were preclarified at 200,000g before the experiment. In a 100-µL reaction volume, 3.0 µM of the polymerized actin was incubated with 0.5 µM of GST-MRH2^649-1051^, AtFIM1, and GST, respectively, in 1× KMEI-buffer (50 mM KCl, 1 mM MgCl_2_, 1 mM EGTA, and 10 mM imidazole-HCl, pH 7.0). 3.0 µM of the polymerized actin alone, and 0.5 µM of GST-MRH2^649-1051^ alone, AtFIM1 alone, or GST alone were also used for the controls. After 1 h-incubation at 22°C, samples were centrifuged at 200,000 g for 1 h in a TL-100 centrifuge (Beckman, Palo Alto, CA, USA) at 4°C. The resulting supernatant was then transferred, and the protein loading buffer was added to the supernatant and the pellet. Equal amounts of the pellet and supernatant samples were separated by 12.5% SDS-PAGE and stained with Coomassie Brilliant Blue R. The band intensities were quantified by densitometry using Image J software (V.1.3; http://rsb.info.nih.gov/ij), and after the background subtraction, the percentage of proteins in the pellet was calculated.

For *in vitro* binding of GST-MRH2^1-449^ and GST-MRH2^649-1051^ to MT, a high-speed co-sedimentation assay, as described elsewhere [62] and similar to that described above, was performed to determine their binding to MT. Porcine brain was used as the MT source, and MT was purified according to a published protocol [63]. GST-MAP65-1 [62], a generous gift of Mao Tonglin (China Agricultural University), was used as a positive control. The reaction volume was 100 µL, containing GST-MAP65-1, GST, GST-MRH2^1-449^, or GST-MARH_2_^1-449^ either in the presence or absence of MT, all in the PEM buffer (1 mM MgCl_2_, 1 mM EGTA, and 100 mM PIPES-KOH, pH 6.9) containing 1 mM GTP and 20 µM taxol. Ten µM AMP-PNP was added for the binding to MT. After 30 min of incubation at room temperature in darkness, samples were centrifuged at 52,000 g in the Allegan 64R centrifuge with a Beckman F1202 rotor (Beckman, Palo Alto, CA, USA) for 15 min. Equal amounts of the pellet and the supernatant samples were separated and visualized as described above.

## Supporting Information

Table S1 Primers used in PCR reactions.
(0.04 MB DOC)

Figure S1 AF organization in wild-type (Col), *mrh2-3*, CA1-1 and *mrh2-3* CA1-1 root hairs. Representative GFP images for various genotypes that contain the 35S:ABD2-GFP construct were shown. Images were projections of about 50 confocal sections, separated by 1 µm distance. The bar represents 20 µm.
(2.11 MB TIF)
