# Supplementary material for: A Mutation in MRH2 Kinesin Enhances the Root Hair Tip Growth Defect Caused by Constitutively Activated ROP2 Small GTPase in Arabidopsis
Source: PLoS One. 2007 Oct 24;2(10):e1074. doi: 10.1371/journal.pone.0001074 (PMC2031828; doi:10.1371/journal.pone.0001074)
Supplement: Table S1 — Primers used in PCR reactions. (0.04 MB DOC) [file pone.0001074.s001.doc]

**Table S1.** Primers used in PCR reactions.

| Name of markers/genes | Name of primers | Primer sequences (5'→3') |
| --- | --- | --- |
| 470805 | T5N23M1S | CGGAAATACCCATTGTTGATAG |
|  | T5N23M1A | GTCAAGACACAAACTAAAGGCA |
| 476269 | T22E16M1S | CTTAGGCTTGGAATACAAGTAAG |
|  | T22E16M1A | GATGGAGCATATATGTAGGATC |
| *MRH2* | YZP77 | CAGGATCCTAATGAGTTCGTCAAATTCCTCC |
|  | YZP79RR | GCTCTAGATCTTGTTCAGCTTGAGAAGTAAG |
|  | MRH2-3S | CTTCGATTTCCGTCGAGATCTC |
|  | MRH2-3A | GTCAGTTCCTTCAGGAAAACAG |
|  | YZP74 | CGGAATTCTCAAGAGATGATCGACCTTTCTC |
|  | YZP76 | CGTACCATGGTAGCTGGAGTCTTCACGAAGC |
|  | YZP105 | CAGGATCCGCTCGGTCTAACGCTGCAGAAG |
|  | YZP114 | ATTCTAGAcATGAGTTCGTCAAATTCCTC |
|  | YZP115 | AGCTCGAGTCATTCCTTAAGCTTTACCATGT |
| *MAP4* | YZP71 | TAAGATCTCCCGGCAAGAAGAAGCAAAGGC |
|  | YZP72 | CTCTAGACCTCCTGCAGGAAAGTGGCCA |
