# Supplementary figures and images for: A Mutation in MRH2 Kinesin Enhances the Root Hair Tip Growth Defect Caused by Constitutively Activated ROP2 Small GTPase in Arabidopsis
Source: PLoS One. 2007 Oct 24;2(10):e1074. doi: 10.1371/journal.pone.0001074 (PMC2031828; doi:10.1371/journal.pone.0001074)

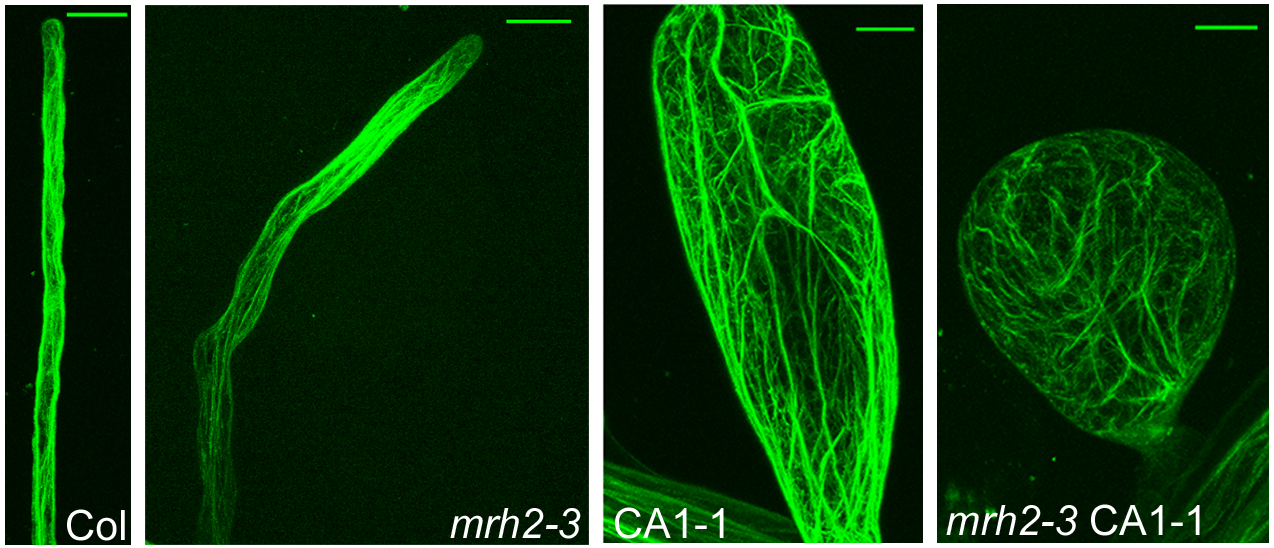

Supplement: Figure S1 — AF organization in wild-type (Col), mrh2-3, CA1-1 and mrh2-3 CA1-1 root hairs. Representative GFP images for various genotypes that contain the 35S:ABD2-GFP construct were shown. Images were projections of about 50 confocal sections, separated by 1 µm distance. The bar represents 20 µm. (2.11 MB TIF) [file pone.0001074.s002.tif]
